# Global health and climate benefits from walking and cycling infrastructure

**DOI:** 10.1073/pnas.2422334122

**Published:** 2025-06-09

**Authors:** Adam Millard-Ball, Monisha Reginald, Yasmina Yusuf, Christopher Bian

**Affiliations:** ^a^Luskin School of Public Affairs, University of California, Los Angeles, CA 90095; ^b^Google, Mountain View, CA 94043

**Keywords:** walking, cycling, transportation policy, street design, climate mitigation

## Abstract

We use a globally consistent dataset—Google Environmental Insights Explorer—to quantify how urban form and transport infrastructure affect walking and cycling rates in 11,587 cities from 121 countries across six continents. At the city level, population density has the largest effect on walk mode share and has a positive impact on cycling too, as higher densities bring destinations within feasible walk and cycle distances. Density has the strongest effects in wealthier countries where travelers often have more travel choices, while in lower-income places, walking and cycling may be primarily driven by financial constraints. We also find positive effects of bicycle lanes (proxying for a wider array of street design practices) on both walking and cycling. In the median city, each new km of bicycle lanes is associated with ~13,400 additional km yr^-1^ of bicycle travel. If every city increased the extent of its bicycle network to the level of Copenhagen, Denmark, our simulations indicate reductions in private vehicle emissions of ~6% and health benefits of ~US$435 billion per year.

## Significance

Walking and cycling emit virtually no pollution and help people integrate physical activity into their daily lives. We demonstrate the untapped potential of street redesigns and urban densification to increase walking and cycling, with measurable benefits for public health and climate change at the global scale. We show that harsh winter or summer climates are not a major barrier to walking and cycling at the annual level, although steep terrain discourages cycling. While northern European cities are well known for their superb infrastructure that supports active travel, we highlight the alternative model of cities such as Osaka, which features less extensive walking and cycling infrastructure but a network of narrow streets with slow-moving traffic where different road users coexist.

Walking and cycling emit practically no carbon or harmful air pollutants, require minimal space for roadways and parking, and help people integrate physical activity into their daily routines (1). At the individual level, previous studies show that each walking or cycling trip saves ~0.4 to 0.5 kg CO_2_ (2), while in aggregate, walking and cycling could reduce urban transport emissions by between 2 and 10% (3). Health benefits include improved mental well-being and reduced cardiovascular disease (4, 5), and cycling can reduce all-cause mortality by 10 to 11% (6).

The potential for walking and cycling (“active travel”) is demonstrated in cities such as Amsterdam (the Netherlands) and Copenhagen (Denmark), where around half of trips are made by active modes (1, 7). The success of these and other cities in promoting active travel is partly rooted in urban form, such as high population densities, which bring more destinations within walking and cycling distance (8, 9), and urban design that creates street-facing uses such as storefronts (10). High active travel mode shares are also the product of policy and infrastructure decisions to build a network of infrastructure such as separated bicycle lanes and safe pedestrian crossings (1, 7, 9, 11–15) and to restrict car use through parking management and other policies (16).

Studies on the impacts of policies to promote active travel, however, are largely based on experiences in Europe, North America, and other high-income regions. The extent to which results are transferable to other contexts is unclear (9), as is the potential impact of scaling up active transport policies worldwide. For example, do density and active street-facing uses universally promote pedestrian travel, or only in the western cities where most research has focused? Partly, this limitation reflects the nature of walking and cycling data: scarce, fragmented, and inconsistently collected. For example, national travel surveys typically have sample sizes that are too small to analyze behavior at the level of individual cities, while city-level surveys are typically only done by larger, wealthier cities. Census data (in the United States, for example) normally only considers the commute to work. There are a handful of comparative studies between pairs of countries (17, 18), and more extensive comparisons collate data from bicycle counters (15, 19), household travel surveys (7, 20–22), or official city reports (8). However, such studies are limited by the labor-intensive process to compile data across countries, and the more cities that are included, the greater the challenges in terms of methodological consistency and temporal mismatch. And these datasets may suffer from selection bias: Cities that promote walking and cycling are the most likely to measure it.

Here, we use a new aggregate data source—Google Environmental Insights Explorer (EIE)—to analyze the impacts of urban form, street design, and other factors on active travel mode shares in 11,587 cities from 121 countries across six continents for the year 2023. The cities in the EIE dataset have a total population of 1.881 billion, accounting for ~41% of the world’s urban population (*SI Appendix*). EIE draws on aggregated, anonymized, and differentially private Location History data from opted-in users and quantifies the number of trips and the distance traveled by each mode within a given city. A key advantage of EIE for our purposes is its global consistency in methods and the definition of a trip. Although the lack of an authoritative reference dataset makes it impossible to benchmark EIE against the “true” numbers, there is a strong correlation between EIE and other sources of active travel data (*SI Appendix*). We also conduct robustness tests to take account of potential biases from lower levels of smartphone access in lower-income countries (*SI Appendix*). Our data represent a major step forward in both qualitative terms (global consistency and range of city sizes and income levels) and quantitative terms (more than 14 times as many cities as the largest study to date).

## Mode Shares for Active Travel

Walking accounts for ~14.3% of trips in cities in the EIE dataset and cycling a further ~2.1%. As a share of travel (i.e., km traveled), the proportions are ~2.0% for walking and ~0.9% for cycling, reflecting the shorter lengths of trips by active modes. Cars and motorcycles account for ~74.2% of passenger km traveled, and public transportation ~23.0%. Unless otherwise stated, our analysis in the remainder of the paper focuses on the share of kilometers traveled rather than the share of trips because travel is more closely related to societal outcomes such as congestion, pollution, and safety. We use “mode share” to refer to the share of travel, not the share of trips.

**Fig. 1.**
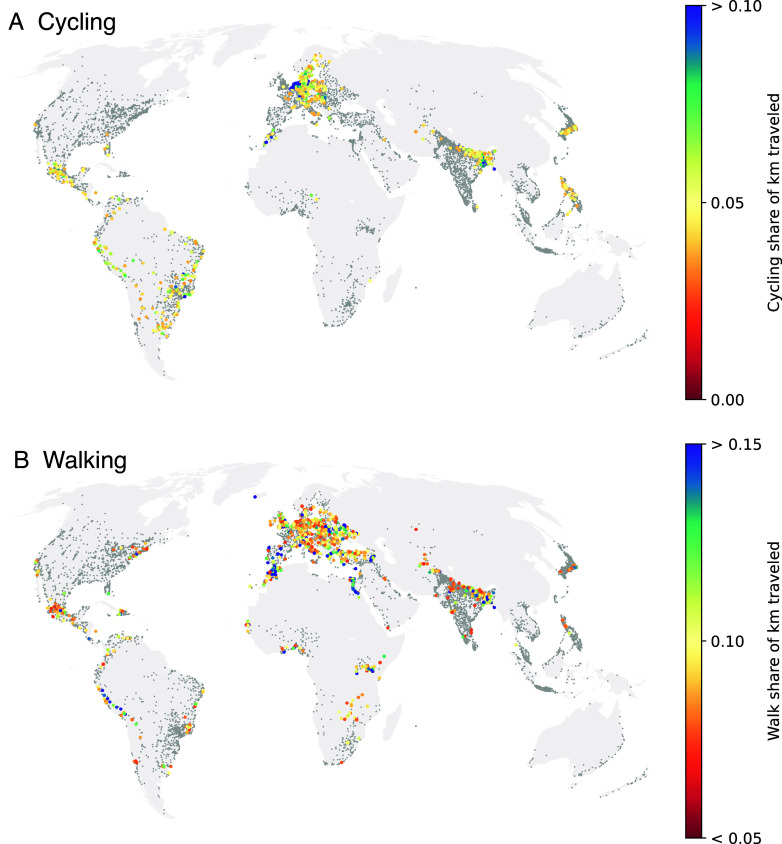
Share of kilometers traveled by bicycle (*A)* and on foot (*B*). The top 10% of cities are highlighted in color; other cities in the EIE dataset are shown in gray.

Northern European cities tend to have the highest bicycling mode shares but clusters of high-cycling cities are also evident in Latin America, Japan, Bangladesh, and Morocco (Figs. 1, *Upper* panel and 2). Cities with high mode shares of walking are also concentrated in Europe, but in eastern and southern European countries such as Slovakia, Serbia, and Belarus that are less well known for their transportation planning and street design efforts (Figs. 1, *Lower* panel and 2). Most of these cities with high walk shares have a low profile in the news media and urban planning literature, even though in our dataset, walking accounts for ~6.9 times as many trips and ~2.3 times as many kilometers as cycling.

**Fig. 2.**
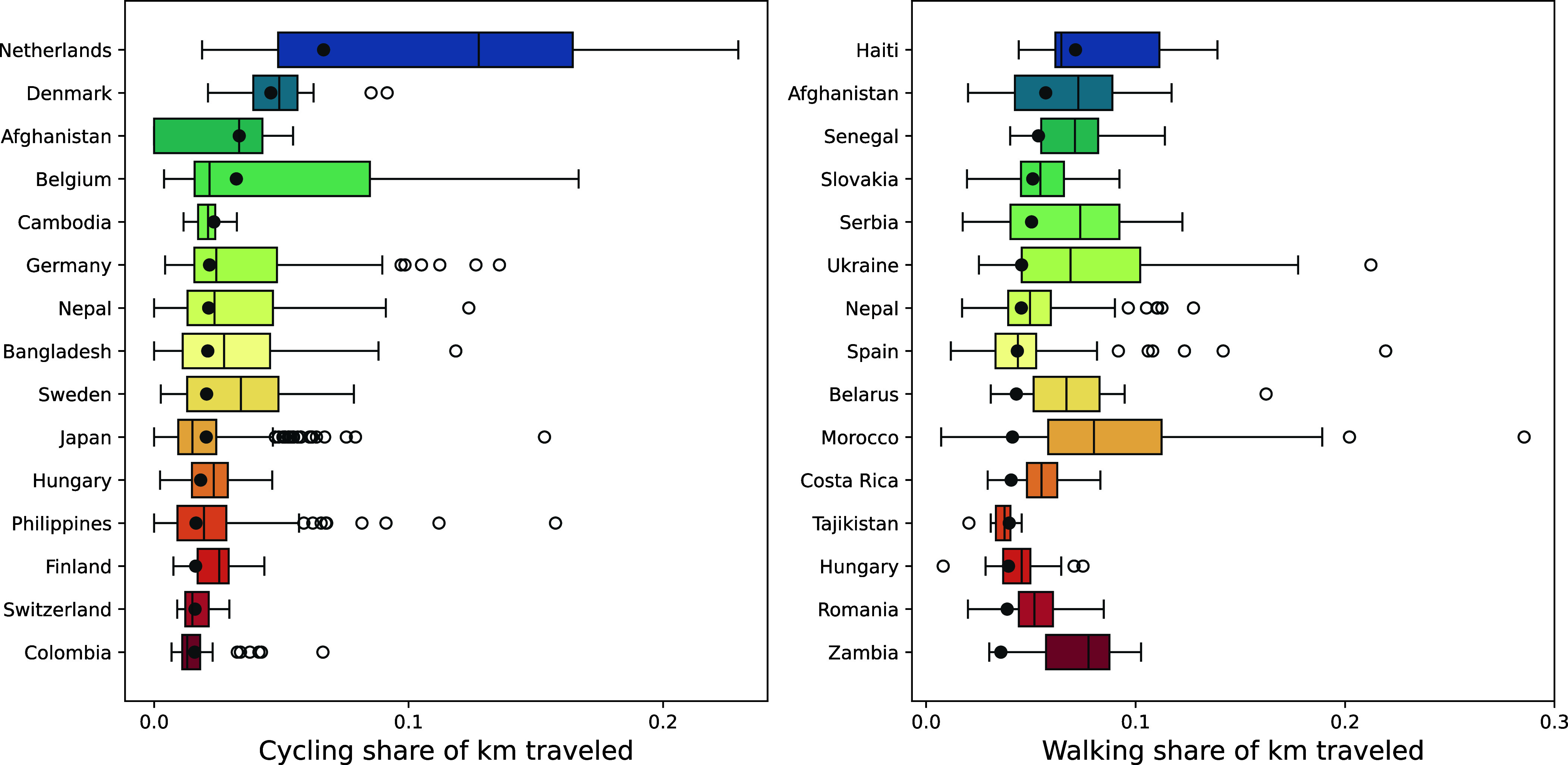
Distribution of city mode share (kilometers). Countries are ordered by the mean share of kilometers traveled by bicycle (*Left*) and on foot (*Right*). For each country, the box shows the distribution of city-level mode shares, with the vertical lines denoting the median city in each country, and the individual dots marking the outliers. The dark gray circle indicates the country-level mean, with cities weighted by distance traveled.

Even within a single country, there is enormous variation in active travel between different cities, reflecting the importance of local-level decisions on infrastructure and land use (Fig. 2). Cycling mode share ranges from virtually zero in thousands of cities to more than one-quarter of kilometers and trips in Dutch cities such as Groningen and Houten. Even in the Netherlands, cycling mode share ranges from ~1.7% (Kerkrade) to ~36.2% (Wageningen). In Japan, the median city has a cycling mode share of ~1.2%, but the outliers, particularly certain suburbs of Tokyo and Osaka, exceed 6%.

Overall, the EIE data back up the well-known bicycling success stories in cities such as Copenhagen and Amsterdam (1, 23). However, they also underscore the importance of walking in achieving high rates of active travel and highlight less well-known examples of cities where active travel plays a major role.

## What Shapes Active Travel?

We use a Bayesian hierarchical model (*SI Appendix*) to estimate country-specific relationships between active travel share and city-level variables, as well as the impacts of country-level factors such as fuel prices. An important caveat is that our results are associational and do not necessarily have a causal interpretation. See the Materials and Methods section and the SI for a further discussion of this issue.

**Fig. 3.**
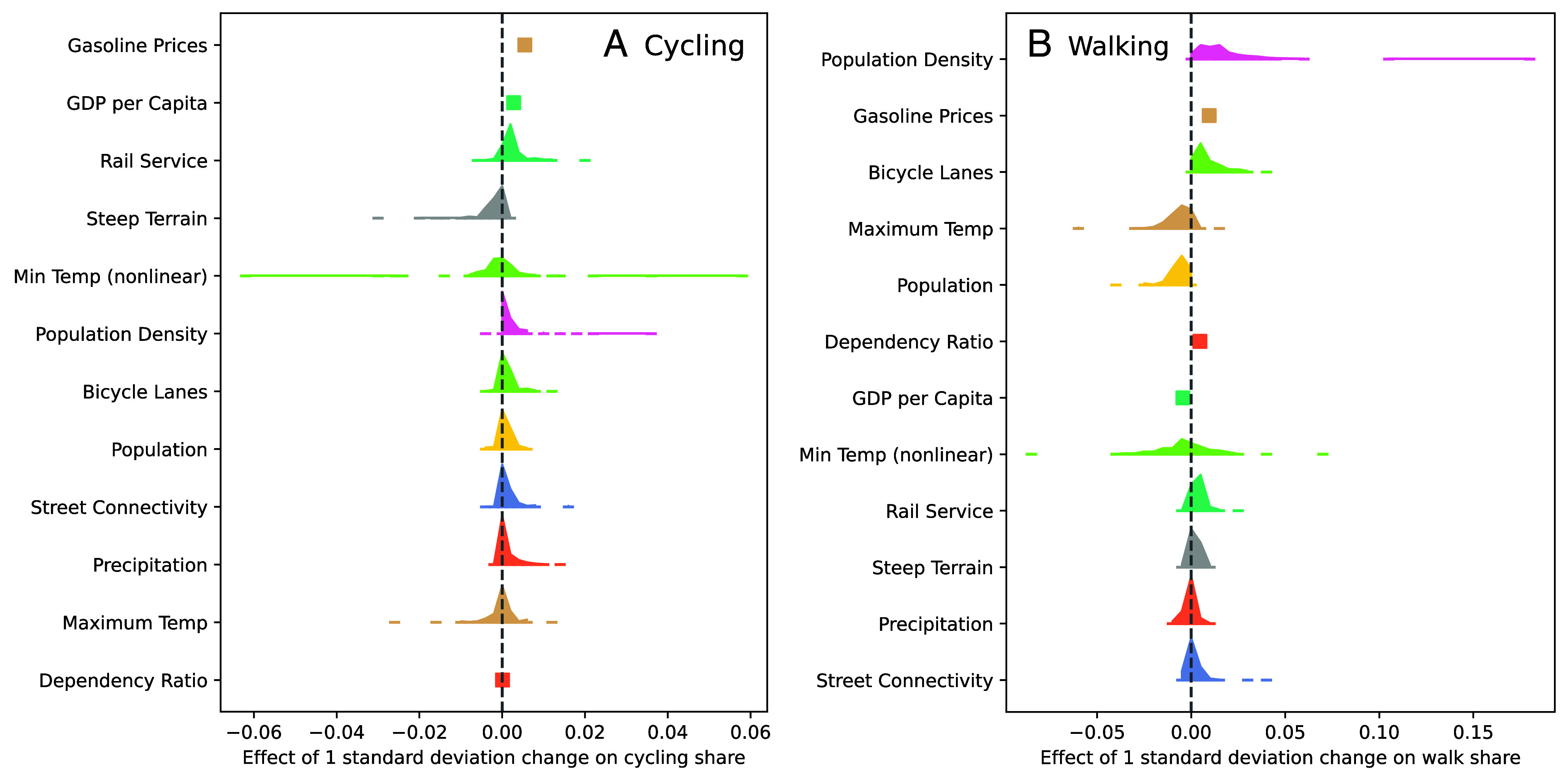
Factors influencing active travel. Each coefficient represents the impact of a one-SD change in each variable on the share of kilometers traveled by cycling (*A*) and walking (*B*). For each city-level variable such as density, the model estimates a separate coefficient for each country. The shaded area shows the distribution of country-level coefficients; for density, for example, the effect on walking ranges from about zero to ~0.11, excluding one outlier. For country-level variables such as gasoline prices, there is only a single coefficient. Variables are ordered by the size of their absolute effect in the median country—density and gasoline prices have the greatest impact on walking and gasoline prices and income (GDP per capita) on cycling. The minimum temperature variable shows the marginal effect of both the linear and squared terms.

Density is the variable with the greatest explanatory power for walk mode share (Fig. 3). This result echoes both theory and empirical evidence—higher density brings destinations closer together, within walking distance (8, 12), and also makes car travel slower and more expensive (24). In our results, a one-SD increase in the natural log of density—moving from the density of San Francisco to that of Taipei—is associated with a ~1.3 percentage point increase in the share of travel by walking in the median country. Higher density is also associated with a greater mode share of cycling (~0.1 percentage points in the median country).

Infrastructure to support active travel, which we measure through the share of roads that have bicycle lanes, is another important factor associated with higher rates of walking. Bicycle lanes are unlikely to directly influence walking, and so our bicycle lanes variable is likely proxying for a wider set of street design practices with which bicycle lanes are correlated, such as sidewalks and crossings. A one-SD increase in the availability of bicycle lanes—moving from the infrastructure of Los Angeles to that of San Francisco—increases the share of travel on foot by ~0.6 percentage points in the median country, and the share of travel by bicycle by ~0.1 percentage points. In the median city, this effect would translate into ~13,400 additional kilometers of bicycle travel annually for each new kilometer of bicycle lanes.

We include physical geography in our model through city-level variables for terrain, minimum and maximum average monthly temperatures, and average annual precipitation. Of all the city-level factors in the model, terrain has one of the largest associations with cycling. Cities with flatter topography have higher bicycle mode shares, again echoing previous research (9). The association between terrain and walking is minimal.

One might expect climate to have a major impact on active travel, and many studies highlight the impact of weather on daily and seasonal travel decisions (9, 25). However, in our model, there is a negligible association between precipitation and the mode shares of walking and cycling. Hotter temperatures decrease walking but have no effect on travel by bicycle. Colder temperatures only reduce walking and cycling in colder climates (*SI Appendix* for further *Discussion*).

At the national level, gasoline price has the largest association with the share of travel by active modes. A one-SD increase in the price per liter (equivalent to US $0.40) is associated with a ~1.0 percentage point increase in walking and a ~0.5 percentage point increase in cycling—notable given that the mode share of cycling across the entire dataset is just ~0.9%. These findings echo a large volume of research that shows how vehicle travel declines in line with rising fuel prices (26). Gasoline prices also have an indirect effect in promoting denser, mixed-use development (27).

Also at the national level, GDP per capita is associated with higher bicycle travel mode share, although the effect is not statistically significant at conventional levels. A one-SD increase in the natural log of GDP per capita, or the equivalent of going from the income levels of India to those of Brazil, is associated with a ~0.3 percentage point increase in bicycle mode share. For walk mode share, the estimated effect of GDP is negative (~–0.4) but also not statistically significant at conventional levels. A null effect is counterintuitive, as much research sees walking as an inferior good that declines as individuals or countries get richer (28, 29).

## Contextual Impacts of Density and Street Design

The hierarchical nature of our model allows the impact of our city-level variables such as density and bicycle lanes to vary by country. The variation in the size of the effect across countries is illustrated by the shaded areas in Fig. 3—the more elongated the area, the more that the effects of that variable differ across countries.

The effect of density on walking is largest in European and north African countries such as Morocco, Germany, France, and Egypt (*SI Appendix*, Figs. SI–6). In Morocco, a one-SD increase is associated with the share of travel by walking rising by ~4.7%, and in Germany, by ~4.0%. For cycling, the effect of density is more muted, except in Europe and in Asian countries such as Japan, the Philippines, and Bangladesh.

**Fig. 4.**
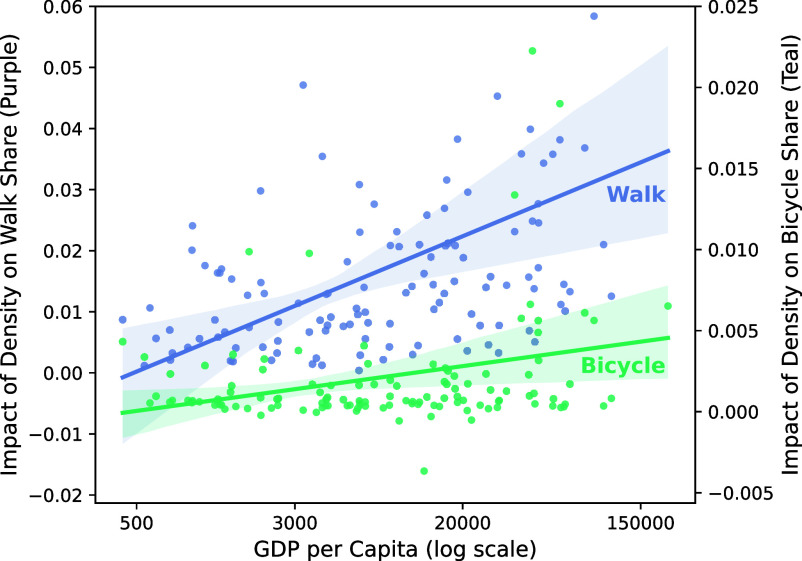
Effects of density vs GDP per capita. For walking trips (purple line), the effects of density are greater in higher-income countries. For cycling (green line), the effect is more muted. Each dot indicates a country-specific coefficient. The shaded areas indicate the 95% CI from the regressions of mode shares against GDP per capita. For clarity, the y axes are truncated to omit outliers.

Our hierarchical model also allows us to analyze how national-level variables such as GDP moderate the impacts of city-level variables such as density. In countries with higher GDP per capita, density has a stronger association with walking rates (Fig. 4). These heterogeneous impacts reinforce and generalize previous findings: Research in North America and Europe highlights the importance of density on walk mode share (12), but studies in lower-income countries such as Colombia (28) and India (30) find muted effects. For cycling rates, the effect of density also increases as GDP per capita rises, but this effect is less than for walking.

A similar analysis shows how the impacts of bicycle lanes vary across countries (*SI Appendix*, Figs. SI–7). Bicycle lanes have a strong effect on walking, but more so in countries in Africa and South Asia, such as Egypt, Rwanda, and Afghanistan. One possible explanation (although speculative in the absence of data) is that these countries may have more cross-city variation in street design, whereas most large cities in Western Europe have near-universal sidewalk provision and other infrastructure to facilitate walking. In terms of cycling rates, bicycle lanes have the strongest effects in Europe, Latin America, and Japan. In Japan, one possibility is that bicycle lanes on arterial roadways play an important role in connecting the narrow, low-speed local streets that are typical of cities such as Tokyo (31).

## Climate and Health Impacts of Street Redesigns

We now consider the potential for redesigned streets to reduce motor vehicle travel and emissions through shifting travel to active modes and to improve health through increasing physical activity. We model the impact of redesigning streets through our country-specific coefficients for bicycle facilities, which serve as a proxy for pedestrian- and bicycle-friendly street design. Full details are in the *SI Appendix*.

**Fig. 5.**
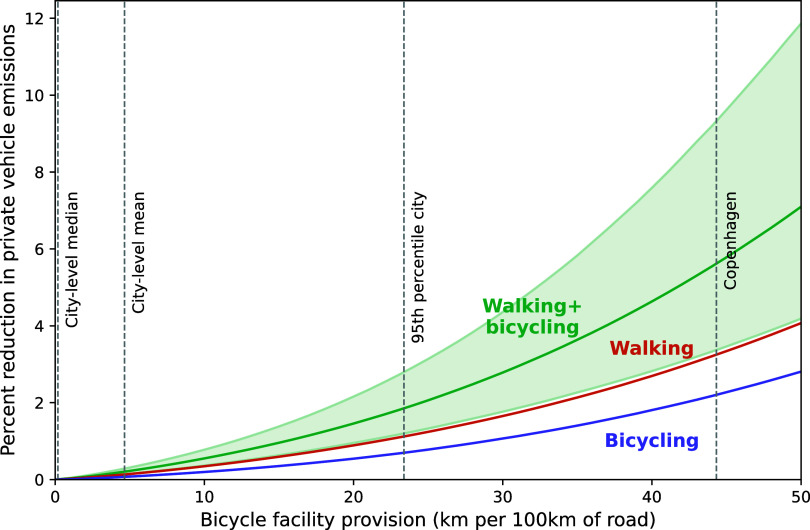
Greenhouse gas benefits of bicycle facilities. For each city, we estimate the impacts of increasing bicycle infrastructure to *B =* 1, 2…50 km of bicycle facility per 100 km of road. Cities that already exceed that level of *B* are assumed not to change (i.e., *B* is a minimum level of provision). The shaded area shows the 95% uncertainty interval.

Fig. 5 shows the estimated reduction in CO_2_ emissions from private vehicles (automobiles and motorcycles) in the cities in our dataset. If every city redesigned streets to increase its provision of bicycle lanes to at least the level of Copenhagen (~44.3 km of bicycle facility for every 100 km of road), we estimate that walking would increase by ~358 billion km per year and cycling by ~305 billion km per year in the cities in our dataset [95 percent uncertainty interval (UI): 206 bn to 604 bn km for walking and 111 bn to 749 bn km for cycling]. The consequent displacement of automobile and motorcycle travel would reduce carbon emissions from private motor vehicles by ~5.6% (95 percent UI: 3.4% to 9.4%), as shown in Fig. 5. The majority of these emissions reductions (~60%) are due to the increased mode share of walking. There would also be substantial benefits from increased physical activity. Using a simple dollar multiplier per kilometer of active travel based on (32), these health benefits amount to US$435 billion per year (95% UI: $218 bn to $851 bn) in 2023 US dollars. As noted above, our dataset accounts for ~41% of the global urban population, and so a first approximation is that these benefits would more than double if extrapolated worldwide.

This “Copenhagen” scenario would entail the construction of ~2.5 million km of new bicycle facilities in the cities in our dataset, on top of the existing network of ~0.3 million km. Fig. 5 also shows the estimated impact of alternative scenarios with lower levels of bicycle lane provision. For example, if all cities grew their bicycle facility provision to at least the level of the 95th percentile city (that of Minneapolis), increased active travel would reduce private motor vehicle emissions by ~1.9% (95% UI: 1.2% to 2.8%) and bring health benefits of US$148 billion (95% UI: $79 bn to $255 bn).

The estimated emission reductions are sensitive to assumptions on how much private motor vehicle travel would be displaced. Our central assumption is that each additional km of active travel displaces 1 km of motorized travel (cars, motorcycles, and public transportation). Substituting alternative displacement ratios which are grounded in the literature generally increases the estimated impact and yields a range of emission reductions of ~4.9 to ~11.9% under the Copenhagen scenario (*SI Appendix*). Under these alternative displacement ratios, each km of bicycling displaces 0.8 to 2.1 km of motorized travel and each km of walking 1.0 to 3.1 km.

## Conclusions

Our results, based on the largest cross-national comparative study to date, suggest two major interventions that local governments can take to promote walking and cycling. First, they can make cities denser, for example through relaxing height limits, parking requirements, and other land-use regulations. We show that density is most important in wealthier countries. In lower-income places, walking and cycling may be driven less by urban form and more by financial constraints and concerns around safety. Thus, promoting density can be seen as a long-term strategy to promote active travel as a country grows richer.

Second, cities can redesign streets to make active travel safer and more comfortable. Our dataset highlights the role of bicycle lanes and paths, but other aspects of street design—sidewalks, safe crossings, and traffic calming measures such as raised intersections—are also important for active travel (12, 33). The quality of the infrastructure is significant too: bicycle lanes that are protected by curbs or parked cars can attract cyclists of all ages and ability levels (23, 34), while express bicycle networks such as those in Montreal provide higher design standards on key routes. Globally, 42% of households own a bicycle (35) and so the question for policymakers is how to encourage more usage rather than increase ownership.

In contrast to medium- and long-term land use changes, street redesign and other active travel infrastructure can be implemented rapidly. In the early stages of the COVID-19 pandemic, cities introduced “pop up” bicycle lanes within weeks or months (36). Our results suggest that the environmental and health gains can be considerable—at the upper end of the range, our “Copenhagen” scenario shows reductions in private vehicle emissions of ~5.6% and health benefits of US$435 billion per year in the cities in our dataset alone. These estimates do not account for nonlinear uptake dynamics, for example, via positive feedbacks through social norms or the “safety in numbers” effect (37). Moreover, climate and physical activity are only two of the benefits of street redesigns that promote active travel. Especially in countries such as India and the United States where pedestrian fatality rates are high and/or rising rapidly, reduced road traffic deaths may be the most significant benefit from bicycle and pedestrian infrastructure. Reduced air pollution and psychophysiological stress are others (1, 5).

While we focus on city-level policy levers, our results also show that policy at the national level—most significantly, higher gasoline prices—matters for active travel. This finding also suggests that a broader set of price incentives (such as tolls and parking charges) can lead to shifts in travel away from cars and toward walking and cycling. These alternative strategies for raising the cost of driving may become increasingly important as the salience of gasoline taxes wanes as electric vehicles gain market share.

The wider message for policymakers is that there are many case studies from which to seek inspiration. Copenhagen and Amsterdam may resonate with some mayors and city transport planners, but differences in culture, planning laws, and resources have historically hampered the uptake of Dutch planning innovations in other countries (38). At the same time, other cities in many parts of the world—Osaka, Japan, and Buenos Aires, Argentina, for example—have achieved high active travel mode shares with less fanfare. And cities such as Osaka highlight the potential of an alternative pathway with less extensive walking and cycling infrastructure, but a network of narrow streets with slow-moving traffic where different road users coexist. Danish and Dutch cities are well known for their superb infrastructure that supports active travel, and their policies and design practices are well documented in news accounts and the academic literature (23, 39). Our results show that this reputation is well deserved but that cities can also look to a wider range of role models for inspiration.

## Materials and Methods

Our dependent variables in the hierarchical regression are mode shares – the shares of distance traveled by i) walking and ii) cycling. Wheelchair use is generally included under “walking” and scooter riding as either walking or cycling depending on travel speed and position in the roadway. We exclude public transportation from the denominator of these mode share calculations as our interest is the substitution between active travel and private motor vehicles. From an environmental and social perspective, substitution of public transportation trips with walking or cycling offers fewer benefits than substitution of car and motorcycle trips. (Separate from our regressions, the descriptive statistics in the “*Mode shares for active travel*” section do include public transportation in the denominator.) Note that access trips to public transportation are represented as separate trips in our data, and so we capture walking and cycling to and from bus stops and rail stations.

We calculated walking and cycling mode shares from Google EIE data. The EIE data are aggregated to Google-defined cities for the year 2023 (no monthly or higher-resolution temporal breakdown is available) and consist of the distance traveled and the number of trips made by each mode. For all cities, the data include trips within the city boundary. For ~30.1% of cities, in- and out-bound trips are also reported (i.e., trips that originate within the city but are traveling to a destination outside or vice versa). We included 50% of the distance of these in- and out-bound trips in our calculations, in line with established greenhouse gas reporting protocols (e.g., 40, p. 79). Results are very similar when we exclude cities that do not have data on in- and out-bound trips (*SI Appendix*).

The cities in our dataset have a total population of ~1.88 billion, according to Google EIE estimates aggregated from the WorldPop dataset. For comparison, the 2025 vintage of the World Bank’s World Development Indicators (WDI) reports a global urban population of ~4.60 billion in 2023. While the urban areas used in the WDI data do not precisely match the extent of the cities covered by EIE, an approximate estimate is that our data cover ~41% of the world’s urban population, with China as the largest missing country. Within just the countries where Google EIE has at least partial coverage, our data cover ~60% of the urban population.

We included independent (predictor) variables in the model (*SI Appendix*, Table SI-1) based on two criteria: i) previous research demonstrating their explanatory power for walking and cycling and ii) the existence of a consistent data source for all the cities in our analysis. Smaller-scale research studies that are restricted to a single city or country have often shown the importance of factors such as accessibility to wheelchair users and other people with disabilities, mixed land uses (also known as land use diversity), accessibility by public transport, walking, and cycling, car parking costs, sidewalk length and quality, and road surface quality (e.g., 41), but data constraints preclude their consideration in our global-scale analysis. Standardized public transport data formats, for example, often lack coverage in the global South, particularly for informal transit systems (42). Another constraint is our units of analysis: Our data are aggregated to cities and the year 2023, and so we cannot capture individual-level attributes, seasonal and weather-related variation, or attitudes and social norms (9, 20).

We used a Bayesian hierarchical model with a beta distribution and an inverse logit link function to assess the association between our independent variables and walking and cycling shares. The hierarchical model allows us to capture the effect of national-level variables (gasoline prices, GDP, and dependency ratio). However, it also allows the effect of city-level variables to vary by country and to be moderated by national-level variables. For example, bicycle lanes or temperature might have a different impact in low-income countries, or in countries with higher gasoline prices. The beta regression model constrains the dependent variable to the interval (0, 1) and is widely used for modeling fractional shares. More details are provided in the *SI Appendix*.

Our hierarchical regressions provide associational evidence and thus the extent to which they reflect causal relationships is uncertain. Causal inference techniques such as instrumental variables are challenging in the global context, and for our purposes, we find their statistical power is limited (*SI Appendix*). However, previous studies of the impact of population density in the United States (43) and France (44) have found little difference between the estimates from ordinary least squares and instrumental variables regressions.

## Supplementary Material

Appendix 01 (PDF)

## Data Availability

Proprietary travel data on walking and bicycling activity were provided by Google Environmental Insights Explorer under a data licensing agreement to UCLA. To request data access, visit https://insights.sustainability.google and click on “Sign up to access” in the upper right-hand corner of the main page and follow prompts. Select “Consultant or NGO working with city government” and please enter “Assessing UCLA research on cycling and walking” when prompted for additional information. A sample of the data for select cities is available publicly. All other data and code are provided in the following repository (45).
