# Supplementary material for: Global health and climate benefits from walking and cycling infrastructure
Source: Proc Natl Acad Sci U S A. 2025 Jun 9;122(24):e2422334122. doi: 10.1073/pnas.2422334122 (PMC12184402; doi:10.1073/pnas.2422334122)
Supplement: Supplementary file 1 — Appendix 01 (PDF) [file pnas.2422334122.sapp.pdf]

Global health and climate benefits from walking and cycling infrastructure

Supplementary Information

May 2025

Authors: Adam Millard-Ball[1*], Monisha Reginald[1], Yasmina Yusuf[1], Christopher Bian[2]

Affiliations:

[1] UCLA Luskin School of Public Affairs, Los Angeles, CA, United States

[2] Google, Mountain View, CA, United States

*Corresponding author. Email: adammb@ucla.edu

Variables and Data

EIE travel data

Environmental Insights Explorer (EIE) draws on aggregated and differentially private geolocation data from Google Maps and Android users who have opted in to sharing their location history. One natural question is the validity and representativeness of the data – does it accurately reflect the travel of people in each city, and in a consistent way around the world? Unfortunately, there is no comparison dataset, authoritative or not, with comparable global coverage. Even for individual cities and countries, data are patchy and inconsistently collected. Pedestrian data in particular are subject to underreporting (respondents may not recall all of their trips on foot) and other biases (*1*).

We therefore compare our EIE data to partial reference sources – the US census, and data reported by Buehler and Pucher (*2*) and Lee et al. (*3*). The US census provides data for commute trips only, but has the advantage of consistency and includes all cities in the country, thus avoiding selection bias. Buehler and Pucher compiled walking mode share for all trips for 36 European and Japanese cities, providing some international coverage, although with a Western bias, and Lee et al. compiled cycling mode-share for all trips for 46 cities across North and South America, Asia, Europe, and Oceania, providing greater international coverage (although still excluding cities within Africa).

Fig. SI-1 shows the comparison. Overall, the EIE data are highly correlated with these other data sources (Pearson's r ranges between 0.62 and 0.91). The EIE data generally provide similar estimates of walking and lower estimates of cycling. For the US Census, EIE tends to provide higher estimates, which is not surprising as non-commute trips are more likely to be made on foot or by bicycle. Overall, the strong correlation between EIE and independent data sources gives us confidence in the dataset, and it is not possible to determine which provides the more authoritative values. A key advantage of EIE for our purposes, however, is its global consistency in methods, the definition of a trip, and the time period (calendar year 2023 for all cities).

Another potential concern relates to potential biases from lower levels of access to smartphones and/or mobile data plans among lower-income people. While cellphone access is widespread in most countries, the same is not yet true of mobile data access. If EIE data are disproportionately drawn from higher-income people, the share of trips by private motor vehicle may be overestimated. We analyze the robustness of our results to this concern below.

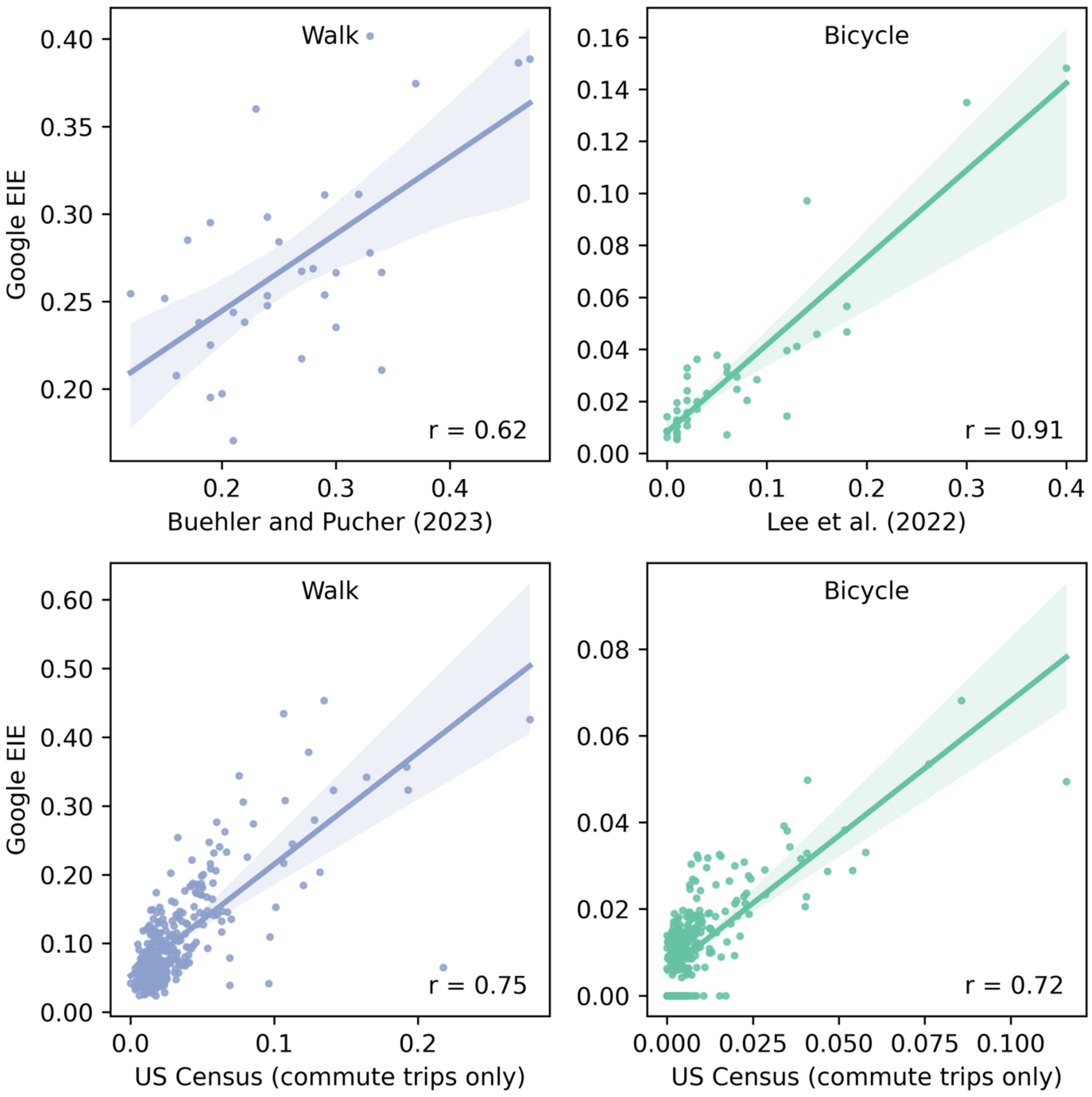

Fig. SI-1. EIE comparison to other data sources. Mode share is shown in terms of share of trips. Left panels: walk trips. Right panels: cycling trips. Upper panels: Cross-national comparisons, for all trips. Lower panels: US comparisons using the 2021 American Community Survey 5-year estimates, for commute trips only. Only cities with >=100,000 population are shown. Shaded areas denote the 95% confidence interval. Pearson's correlation coefficient *r* is indicated.

Independent variables

Table SI-1 summarizes the independent variables that we include in our analysis, along with the underlying data source. The brief rationale for including each variable is as follows:

- **Population density** brings destinations closer together, making walking and cycling feasible for more trips (*3*, *4*), while total population captures scale effects
- **Connected streets** – a gridiron pattern rather than culs-de-sac, for example – provide direct routes and reduce travel distances, enabling more walking and cycling trips (*5*). Street connectivity is sometimes referred to as "design," for example in the commonly used 3Ds or 5Ds framework (*6*).
- **Street design** incorporating sidewalks, bicycle lanes, and pedestrian and bicycle paths helps keep road users safe and encourages active travel (*7*)
- **Public transport** can be complementary to walking and cycling, enabling people to live without a car, and most riders will walk or bike at each end of the bus or train trip (*1*).
- **Motorways** increase the speed of driving, and may lead to people substituting walk or bicycle trips in place of driving
- **The physical geography** of a city – extreme temperatures, rain and snow, and steep slopes – can make it uncomfortable to walk or cycle (*3*, *4*)
- **National-level factors** such as fuel prices and income affect active travel through the affordability of car ownership and use (*3*, *8*), while age structure matters as older adults tend to cycle less (*9*, *10*)

For density, we weight the density of 1 km^2 grid cells by population in order to minimize dependence on arbitrary city boundaries and the presence of water and open space (*11*). Because some previous studies (e.g. *12*) have suggested that density has nonlinear effects (for example, above a certain threshold, the marginal effect of density declines to zero), we include a squared term for our density variable and use it in one of our specifications.

For bicycle lanes, we measure the ratio of bicycle facility length to road length in OpenStreetMap (OSM) road network data. Our road length measure excludes service roads such as alleys and paths for pedestrians and bicyclists. In order to reflect the various ways that bicycle facilities are denoted in OSM (see https://wiki.openstreetmap.org/wiki/Bicycle), we identify bicycle facilities using the tag *highway=cycleway* or *cycleway=**, excluding those tagged as *cycleway=shared_lane* or *cycleway=no*. One uncertainty in our dataset is the completeness of bicycle facility tags in OSM. While the overall road network is complete in most countries (*13*) and a study in Canada shows that OSM bicycle facility data is as good or better than official open-data sources (*14*), it is unclear whether the same is true elsewhere in the world. Because of concerns over the completeness of the underlying data, we do not distinguish between different types of bicycle facilities such as painted lanes vs protected bicycle lanes, even where these are tagged as such in OSM.

Note that a ratio of (say) 0.5 does not mean that half of the roads in a city have bicycle facilities. First, the length of bicycle facilities includes off-road paths such as those through parks and along rivers and rail lines. Second, some roads may have two parallel bicycle paths, one on each side of the road, where these are represented as separate facilities in OSM. Thus, some roads may have 2km of bicycle path for each 1km of road length.

Our climate variables are calculated based on monthly temperature and precipitation data, provided by the European Centre for Medium-Range Weather Forecasts in the form of a 0.25° x 0.25° grid. We assign each city to the grid cell that intersects the city's centroid. Because the effects of climate may be nonlinear—for example, colder temperatures may have a greater effect in colder climates—we include squared terms for temperature.

National-level gasoline prices (2018 vintage) are reported by GIZ (*15*). For seven countries, we interpolate the price based on an earlier vintage (2016) of the price data, or if neither 2016 nor 2018 data were available, based on average prices in the same geographic region.

In addition to the substantive variables shown in Table SI-1 and discussed above, we include a binary variable that controls for whether in- and outbound trips (i.e., trips that have an origin or destination outside the city boundary) are included in the EIE dataset. We include this control because the shares of travel by walking or cycling are generally higher for trips within a city than for trips into or out of a city, given that the former are more likely to be short trips easily accomplished by active modes.

Most variables were log transformed to address skew in the underlying data, as indicated in Table SI-1. We also standardize all of the non-binary variables by subtracting the mean and dividing by the standard deviation.

We aggregate all city-level data to the polygons of the Google-defined cities. While we did not have access to the original polygon boundaries, we approximate them using open-source and similarly licensed materials: OpenStreetMap, national census boundaries, Humanitarian Data Exchange, AmeriGEOSS, and the Global Administrative Areas dataset at www.gadm.org. For each polygon, we verify with Google that our overlap is greater than 90%. Discrepancies between our boundaries and Google's original polygon boundaries generally occur in uninhabited or sparsely inhabited areas, because there is generally consistency in the inclusion of population centers across open-source materials and Google's boundaries. As a result, discrepancies have a minimal effect on aggregated data, which are driven by areas with more roads and population density.

Variable	Source and comments
**City level**	
Weighted population density (natural log)	Calculated using GHS-POP from the Global Human Settlements Layer (GHSL). https://dx.doi.org/10.2760/098587
Population (natural log)	GHS-POP, as above
Street connectivity (SNDi) (natural log)	SNDi is an index of street-network disconnectedness, documented in Barrington-Leigh and Millard-Ball (*16*)
Street-network connectivity added by bicycle lanes and pedestrian paths	Difference between SNDi calculated for the network including versus excluding these lanes and paths, documented in Barrington-Leigh and Millard-Ball (*16*)
Bicycle facilities (km per km of road)	OpenStreetMap, vintage October 2023, downloaded from planet.openstreetmap.org
Rail (binary variable)	1 = EIE reports any rail trips in that city in 2023
Motorways (km per km of road)	OpenStreetMap, vintage October 2023, downloaded from planet.openstreetmap.org
Slope (natural log)	Calculated from the US Geological Survey GMTED 2010 dataset
Monthly maximum temperature	Average temperature in the hottest month of the year. European Centre for Medium-Range Weather Forecasts ERA5 (2018 to 2022)
Monthly minimum temperature	Average temperature in the coldest month of the year. European Centre for Medium-Range Weather Forecasts ERA5 (2018 to 2022)
Annual precipitation (natural log)	European Centre for Medium-Range Weather Forecasts ERA5 (2018 to 2022)
**Country level**	
GDP per capita (natural log)	World Development Indicators (2022 or most recent year)
Dependency ratio	Ratio of working age (15-64) to non-working age population. World Development Indicators (2022 or most recent year)
Gasoline price	GIZ, International Fuel Prices 2018/19 (*15*)

Table SI-1. Data sources

S2. Bayesian hierarchical model

We used a Bayesian hierarchical model to assess the association between our independent variables and walking and cycling shares. The hierarchical model allows us to capture the effect of national-level variables (gasoline prices, GDP, and dependency ratio). But it also allows the effect of city-level variables to vary by country, and to be moderated by national-level variables. For example, bicycle lanes or temperature might have a different impact in low-income countries, or in countries with higher gasoline prices.

Another advantage of the hierarchical model is partial pooling, sometimes called "shrinkage" (*17*). In countries with many cities in our dataset, the model provides country-specific estimates of the impacts of our city-level variables. In countries with more sparse data, the model "shrinks" to the global mean, in effect using information from cities in other countries with similar levels of GDP and gasoline prices.

For each city i in country $j \in \{1, 2, \dots, m\}$ we model the modal share S_{ij} using the beta distribution with an inverse logit link function as in Eq 1.

$$S_{ij} \sim Beta\left(g^{-1}\left(\alpha_j + X_{ij}\beta_j\right), \kappa\right) \quad (1)$$

Where X_{ij} is a vector of city-level variables, g^{-1} is the inverse logit function, α_j is a country-specific intercept, β_j is a country-specific vector of coefficients estimated as in Eq. 2, and κ is the precision.

$$\beta_j \sim \text{multivariate normal}\left(\delta + \gamma Z_j, \Omega\right) \quad (2)$$

Where δ is the intercept, γ, Z_j are vectors of coefficients and country-level variables, and Ω is the variance-covariance matrix.

We estimated the model using the Stan software package (*18*) and the cmdstanpy Python bindings. We used weakly informative priors to help with convergence: *Normal* (0, 1) for the country- and city-level coefficients, the Cholesky factorization of an LKJ covariance matrix with diagonals *Exponential* (1), and *Gamma*(4, 0.2) for the precision κ, similar to the recommendations in the Stan User's Guide (*19*). We used four chains, discarded 1000 warm-up iterations, and used the estimates from the final 1000 iterations per chain. We visually assessed posterior predictive plots (Fig. SI-2) and, to ensure mixing, the coefficient trace plots (Fig. SI-2). We also assess model fit through calculating posterior p-values and the mean squared error of our predicted (fitted) values.

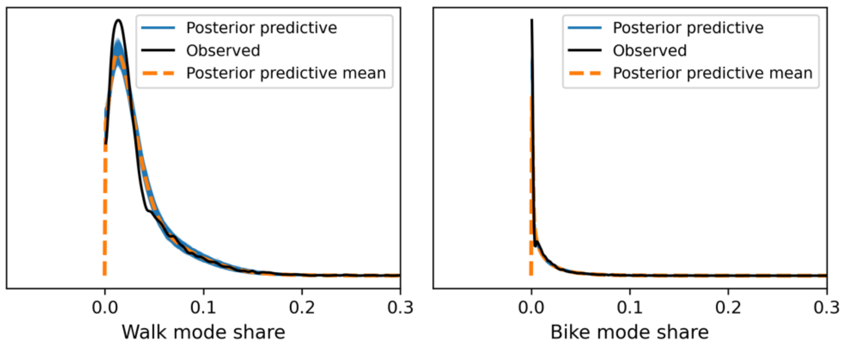

Fig. SI-2. Posterior predictive plots for walking (left) and bicycling (right)

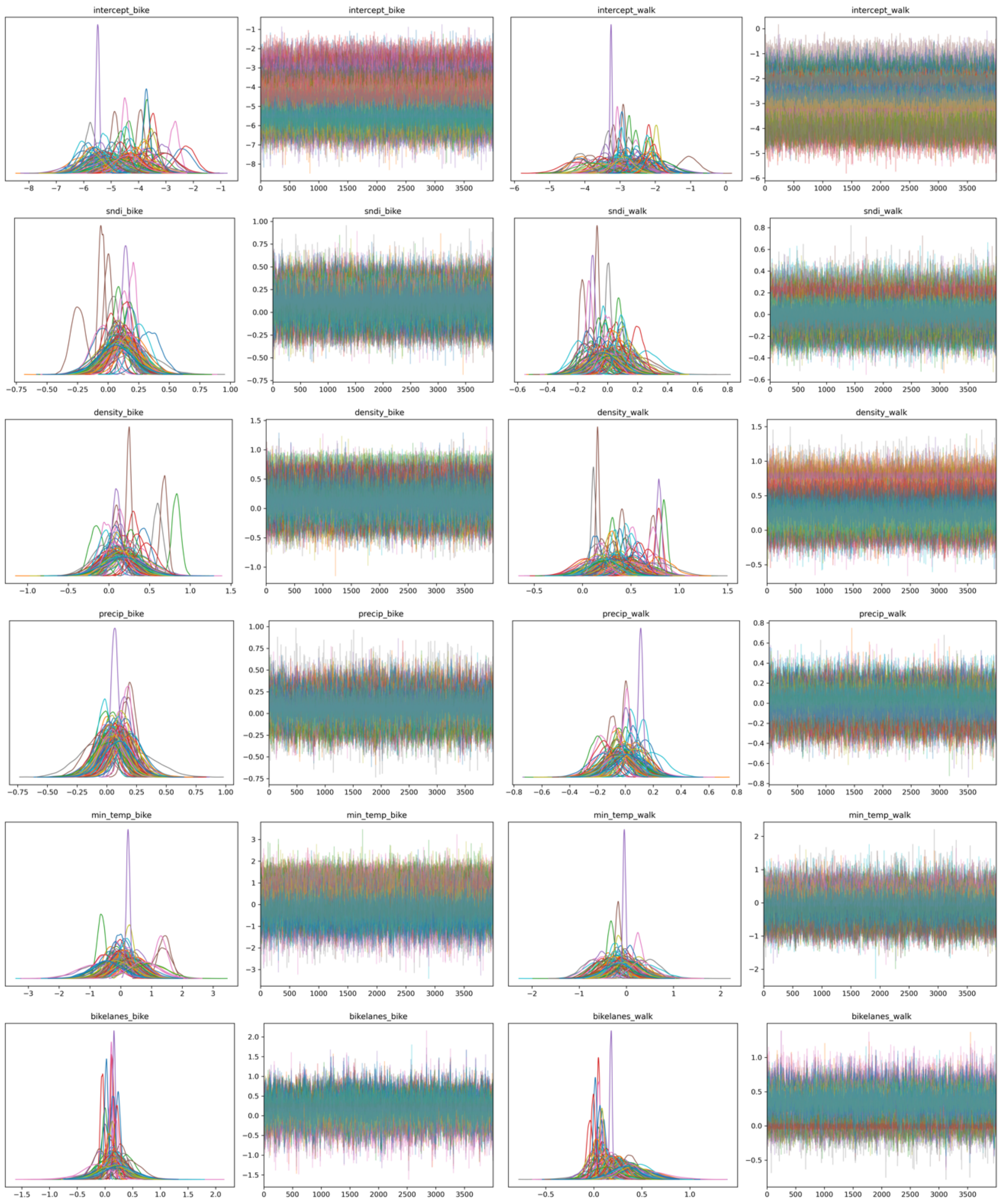

Fig. SI-3. Trace plots. The patterns indicate that the chains are well mixed. Each plot shows a different city-level coefficient, separately estimated for cycling (*right*) and walking (*left*). The left panels show the distribution of coefficient estimates (each color indicates a different country), and the right plots show the traces over the 4,000 iterations. The plots are for the preferred model (see below).

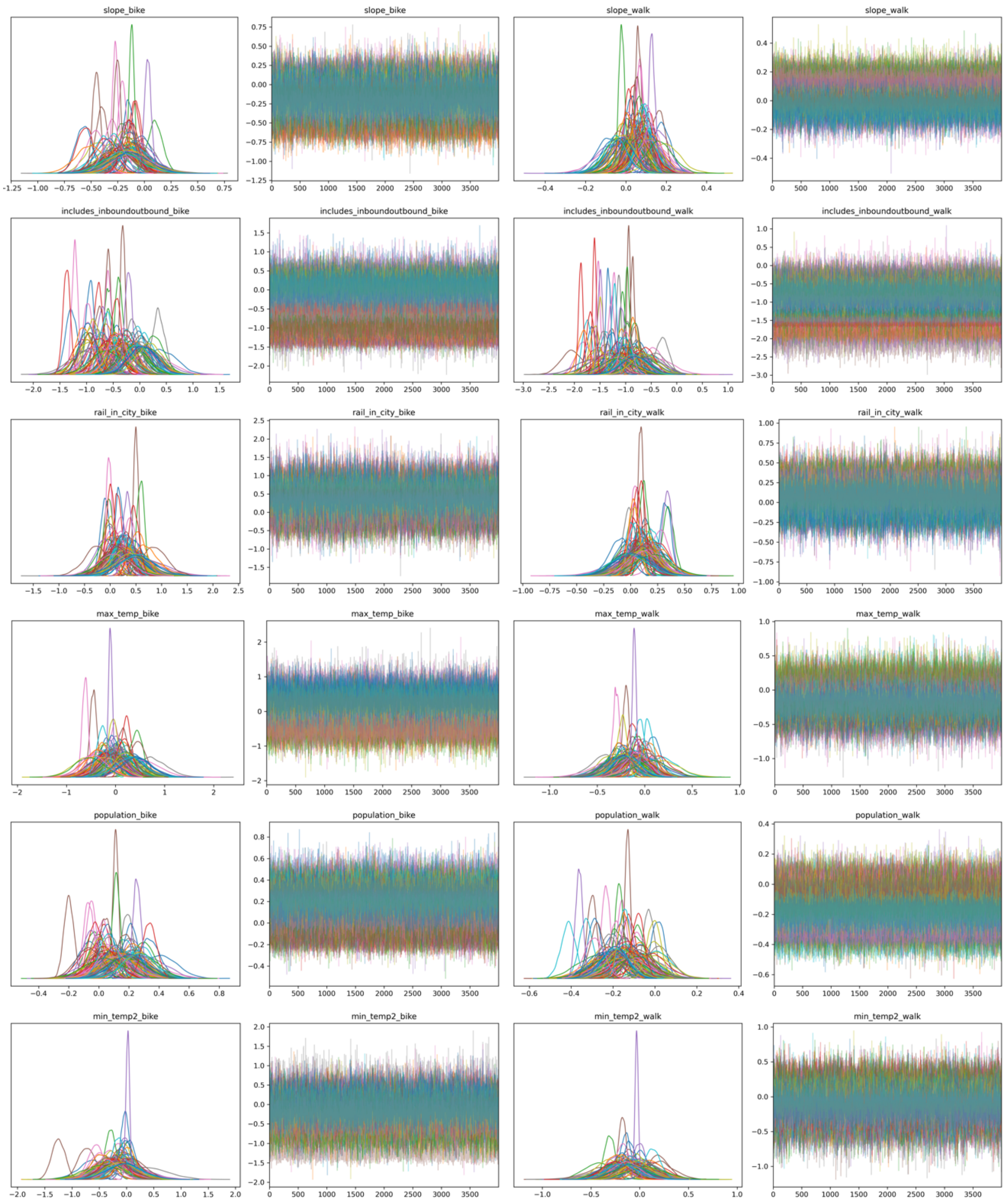

Fig. SI-3. Trace plots (continued)

Alternative specifications

We estimate three models with different subsets of the variables in Table SI-1, all of which use a beta regression model.

- **Preferred**: discussed below and presented in the main text
- **Saturated**: includes all the variables in Table SI-1
- **Nonlinear**: includes all the variables in the preferred model, plus squared terms for density, minimum temperature, maximum temperature, and precipitation

Our preferred model includes all the variables in Table SI-1 except for (i) motorways and (ii) street-network connectivity added by bicycle lanes and pedestrian paths, for which the estimated effects are statistically indistinguishable from zero, and which do not change the interpretation or the signs or magnitudes of other coefficients. Our preferred model also includes a squared term for minimum temperature, but not the other squared terms in the nonlinear model, which also do not change the interpretation or the signs or magnitudes of the estimated effects, but make interpretation more challenging.

We use a beta regression model and an inverse logit link function because our dependent variables—mode shares—are constrained to the [0, 1] interval. We add 0.001 to any observations where the dependent variable has a value of zero, as the beta model cannot take values of 0 or 1. As a robustness test, we estimate a linear model using the same variables, although we prefer the beta model due to potential biases in a linear model, which can generate fitted values below zero or greater than one. Our qualitative conclusions are unchanged (Table SI-2), although the estimated impact of our bicycle lane expansion scenario is somewhat larger when using the beta specification.

Table SI-2 shows the coefficient estimates and uncertainty interval for all four models, and Fig. SI-4 plots the marginal effects of each coefficient. Figs SI-5 through SI-7 provide additional results that complement the plots in the main text.

We also undertake three robustness tests that estimate the beta model using: (i) the share of trips rather than the share of km traveled as the dependent variable; (ii) a subset of the data with only cities that have data for in- and out-bound trips that cross the city boundary, in addition to trips within the city, and (ii) a subset of the data with only cities in countries that are above the median GDP for countries in our sample. The second test explores the sensitivity to potential data incompleteness, while the third addresses the potential concern of underestimating walking and bicycling trips in lower-income countries where smartphone access with mobile data plans may not be widespread. Fig. SI-4 shows the comparison. In the trips model, the estimated effects of variables are often larger than in our other models, likely because the observed share of trips by walking and cycling is larger than the observed share of km travel. For the second and third robustness tests, the models generate very similar results.

Impacts of density

We find that the impact of density declines with GDP (see main text). One possible explanation is that a non-linear relationship with density is creating a spurious relationship with GDP, since lower-income countries generally have high densities. However, the coefficient on GDP is similar when we include a squared term for density in the model (Fig. SI-4 and Table SI-2). This finding does not preclude non-

linear density effects or the existence of a threshold above which density has a more limited influence, as suggested elsewhere in the literature (*20*, *21*). Our model suggests diminishing returns to density because the variable enters the model in log form, and also indicates that any non-linearity varies considerably across countries.

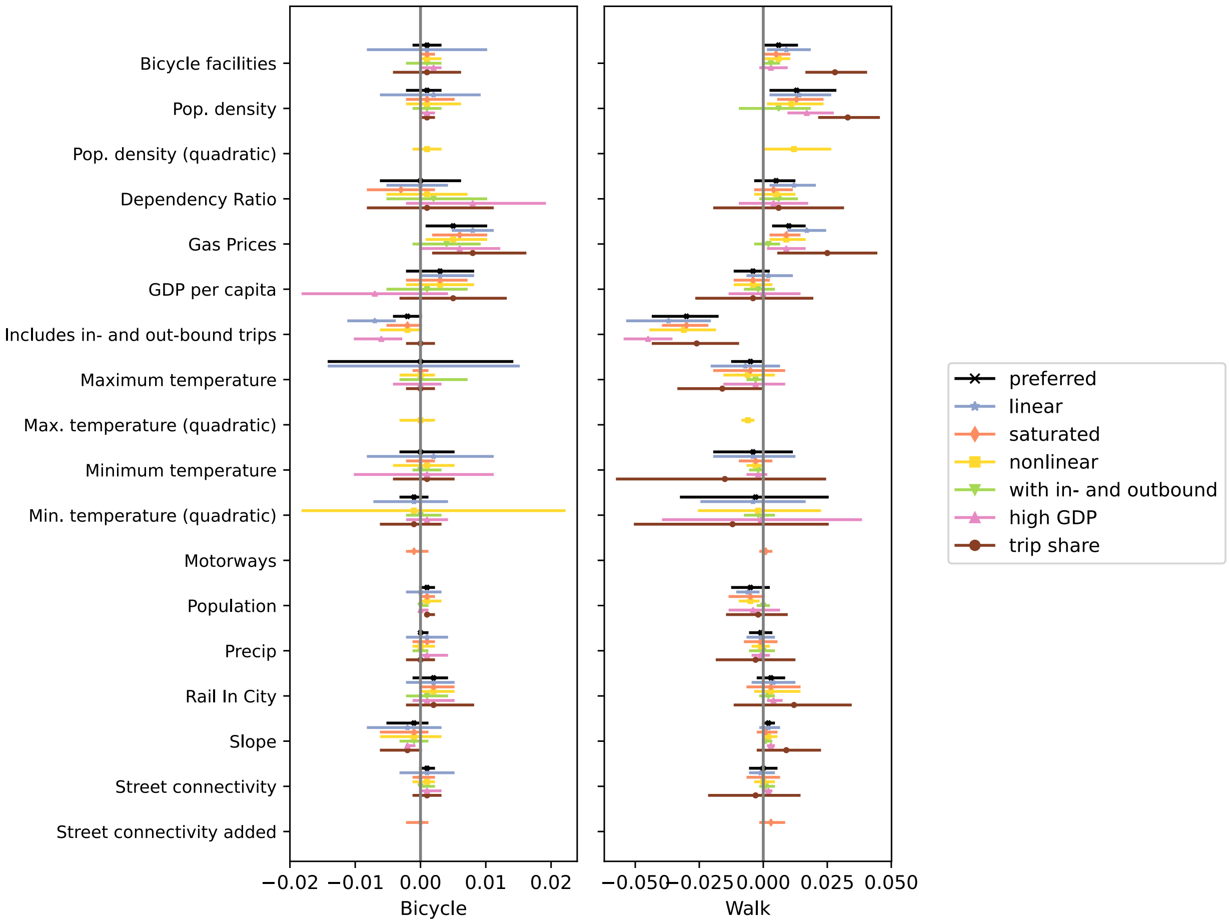

Fig. SI-4. Model comparison. The points indicate the median estimate of the marginal effect for the median country, with the 90% uncertainty interval (UI) for that country indicated by the span of the line. Note that the UI reflects the size of the median country (countries with fewer data points will have a higher UI) as well as the inherent uncertainty in the model. Quadratic terms indicate the marginal effect of the combined linear and squared terms.

	Walking				Bicycling			
	Preferred	Linear	Nonlinear	Saturated	Preferred	Linear	Nonlinear	Saturated
**Country-level variables**								
GDP per capita	-0.004 (-0.011, 0.002)	0.002 (-0.006, 0.011)	-0.004 (-0.011, 0.003)	-0.004 (-0.011, 0.002)	0.003 (-0.002, 0.008)	0.003 (-0.000, 0.008)	0.003 (-0.002, 0.008)	0.003 (-0.002, 0.007)
Gasoline price	0.010 (0.004, 0.016)	0.017 (0.010, 0.024)	0.009 (0.003, 0.016)	0.009 (0.003, 0.014)	0.005 (0.001, 0.010)	0.008 (0.005, 0.011)	0.005 (0.001, 0.010)	0.006 (0.002, 0.010)
Dependency ratio	0.005 (-0.003, 0.012)	0.012 (0.003, 0.020)	0.005 (-0.003, 0.012)	0.004 (-0.003, 0.011)	0.000 (-0.006, 0.006)	-0.000 (-0.005, 0.004)	0.001 (-0.005, 0.007)	-0.003 (-0.008, 0.002
**City-level variables**								
Pop. density	0.013 (0.003, 0.028)	0.014 (0.003, 0.026)		0.013 (0.006, 0.023)	0.001 (-0.002, 0.003)	0.002 (-0.006, 0.009)		0.001 (-0.002, 0.005)
Pop. density (nonlinear)			0.012 (-0.000, 0.026)				0.001 (-0.001, 0.003)	
Population	-0.005 (-0.012, 0.002)	-0.006 (-0.010, -0.002)	-0.005 (-0.009, -0.002)	-0.005 (-0.013, -0.000)	0.001 (0.000, 0.002)	0.000 (-0.002, 0.003)	0.001 (-0.000, 0.003)	0.001 (-0.000, 0.002)
Street connectivity	0.000 (-0.005, 0.005)	-0.001 (-0.005, 0.004)	0.000 (-0.003, 0.004)	0.000 (-0.006, 0.006)	0.001 (-0.000, 0.002)	0.001 (-0.003, 0.005)	0.001 (-0.001, 0.002)	0.000 (-0.001, 0.002)
Street connectivity added*				0.003 (-0.001, 0.008)				-0.000 (-0.002, 0.001)
Bicycle facilities	0.006 (0.001, 0.013)	0.009 (0.002, 0.018)	0.006 (0.001, 0.010)	0.005 (0.000, 0.010)	0.001 (-0.001, 0.003)	0.001 (-0.008, 0.010)	0.001 (-0.000, 0.003)	0.001 (-0.000, 0.002)
Rail	0.003 (-0.002, 0.008)	0.004 (-0.004, 0.012)	0.003 (-0.003, 0.014)	0.003 (-0.006, 0.014)	0.002 (-0.001, 0.004)	0.002 (-0.002, 0.005)	0.002 (0.000, 0.005)	0.002 (-0.000, 0.005)
Motorways				0.001 (-0.001, 0.003)				-0.001 (-0.002, 0.001)
Slope	0.002 (-0.000, 0.004)	0.002 (-0.001, 0.006)	0.002 (-0.000, 0.005)	0.001 (-0.002, 0.005)	-0.001 (-0.005, 0.001)	-0.002 (-0.008, 0.003)	-0.001 (-0.006, 0.003)	-0.001 (-0.006, 0.001)
Maximum temperature	-0.005 (-0.012, -0.001)	-0.007 (-0.020, 0.006)		-0.005 (-0.019, 0.008)	-0.000 (-0.014, 0.014)	0.000 (-0.014, 0.015)		-0.000 (-0.001, 0.001)
Max. temperature (nonlinear)			-0.006 (-0.008, -0.004)				-0.000 (-0.003, 0.002)	
Minimum temperature				-0.003 (-0.009, 0.003)				-0.000 (-0.002, 0.002)
Min. temperature (nonlinear)	-0.003 (-0.032, 0.025)	-0.004 (-0.024, 0.016)	-0.002 (-0.025, 0.022)		-0.001 (-0.003, 0.001)	-0.001 (-0.007, 0.004)	-0.001 (-0.018, 0.022)	
Precipitation	-0.001 (-0.005, 0.003)	-0.001 (-0.006, 0.004)	-0.001 (-0.004, 0.002)	-0.001 (-0.007, 0.005)	0.000 (-0.000, 0.001)	0.001 (-0.002, 0.004)	0.000 (-0.001, 0.002)	0.001 (-0.001, 0.002)
Includes in- and out-bound trips	-0.030 (-0.043, -0.018)	-0.037 (-0.053, -0.021)	-0.031 (-0.044, -0.019)	-0.030 (-0.039, -0.022)	-0.002 (-0.004, -0.000)	-0.007 (-0.011, -0.004)	-0.002 (-0.006, -0.000)	-0.002 (-0.005, -0.000)
**Model performance**								
Root mean squared error	0.016	0.018	0.016	0.016	0.013	0.012	0.013	0.013
Posterior p-value**	0.744	0.504	0.747	0.736	0.592	0.504	0.614	0.596

* Street connectivity added by bicycle and pedestrian paths

** Closer to 0.50 is better.

Table SI-2. Marginal effect estimates. For each model, we show the median estimate (i.e., the median of the 4,000 iterations). The 90% uncertainty interval (UI) is given in parentheses. For the city-level variables, these estimates refer to the median country; thus, the UI reflects the size of the country (countries with fewer data points will have a higher UI) as well as the inherent uncertainty in the model. All variables except for the binary variables are standardized. For the linear model, the marginal effects are the same as the coefficient. Where a variable enters in quadratic form ("nonlinear"), we show the combined marginal effect of both the linear and squared terms. All models also include an intercept at both city and country levels.

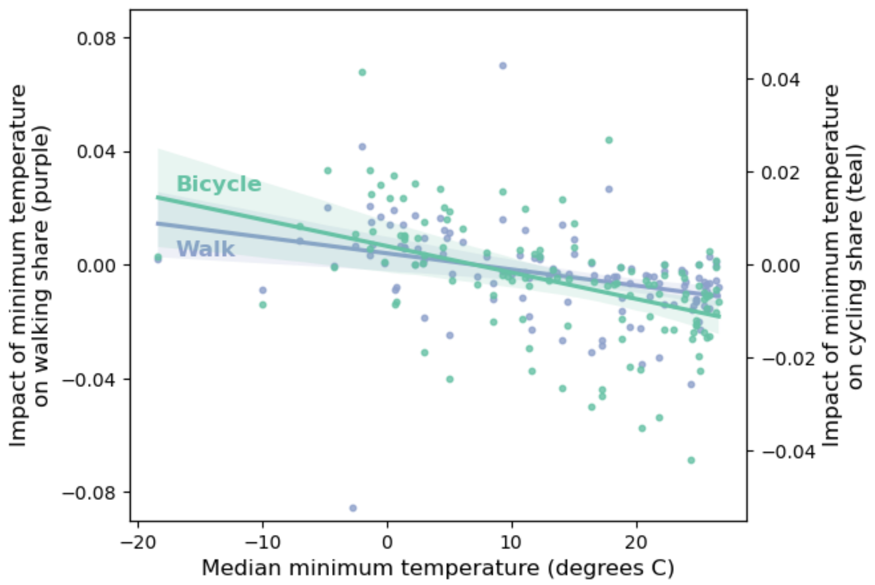

Fig. SI-5. Marginal effects of cold climates on mode share. Each point represents the marginal effect of a one standard deviation increase in the minimum temperature in each country (calculated as the median minimum temperature for cities in our dataset). The marginal effects include both the linear and quadratic term in the model.

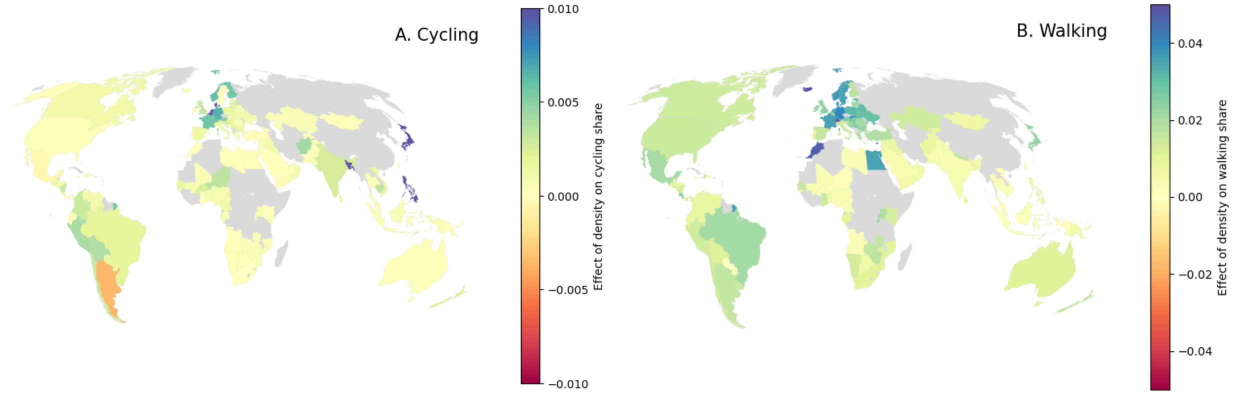

Fig. SI-6. Country-level effects of density on mode share for bicycling (*A*) **and walking** (*B*)

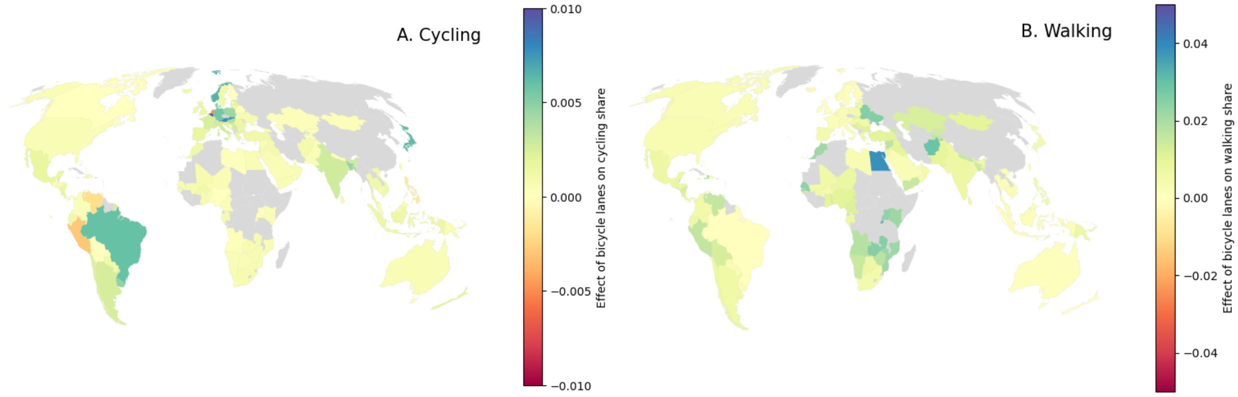

Fig. SI-7. Country-level effects of bicycle lanes on mode share for bicycling (*A*) **and walking** (*B*)

S3. Potential for causal inference via instrumental variables

As noted in the main text, our hierarchical regressions provide associational evidence for the impact of a range of variables on walking and cycling mode share. However, our estimates may suffer from the well-known biases that affect ordinary least squares (OLS) and similar regression models, such as omitted variables and the choice of functional form. A range of causal inference techniques, particularly instrumental variables (IV) (*22*), has therefore been employed in land use and transportation research. For example, Duranton and Turner (*23*) use the presence of aquifers as an instrument for density, on the grounds that aquifers can support lower-density, scattered development that uses well water and thus is not dependent on an (urban) public water supply. Buehler and Pucher (*24*) use city land area and the membership of bicycling advocacy groups as instruments for bicycle lanes.

For *density*, we experimented with several potential instruments—groundwater depth (*25*), seismic risk (*26*), soil type (*27*), and historic (AD 1900) population (*28*). However, groundwater depth has a first-stage correlation with the opposite sign to that expected (deeper groundwater is associated with lower density), historic population is likely a better instrument for current population (which is also in our model) than for density, and the other two instruments (soil type and seismic risk) are weak as evidenced through the Cragg-Donald *F* statistic. Figure SI-8 shows the correlations between density and potential instrumental variables.

For *bicycle lanes*, we experimented with two instruments—the lengths of canals and disused rail lines in European and North American cities, where such infrastructure is commonly converted to bicycle and pedestrian paths. We obtain data on both from OpenStreetMap. However, these instruments are weak, as evidenced through the Cragg-Donald *F* statistic. Data for other potential instruments (e.g. historic bicycle lane network data or the membership of bicycling advocacy groups) are not available at the global level.

In general, our IV estimates are larger than equivalent OLS estimates (Tables SI-3 and SI-4). However, given the challenges discussed above, we consider them to provide little information. The exclusion restrictions that are required for IV are generally easier to satisfy in the context of a single country (as in the studies that are cited above), rather than at the global level where causal processes are likely to be heterogeneous across countries.

Finally, we note that while in principle our estimates for density may suffer biases from self-selection (sorting) and unobserved neighborhood characteristics (e.g., mix of uses), in practice two of the major studies that instrument for density find no meaningful difference between their OLS and IV estimates. For example, Duranton and Turner (*23*) note: "[IV] coefficient estimates are statistically indistinguishable from those in our table of OLS estimations. This suggests that omitted variables correlated with driving and urban form are not causing economically important bias in our estimates of the relationship between urban form and driving." Blaudin de The et al. (*29*, Table 5) also find no major differences between their OLS and IV estimates.

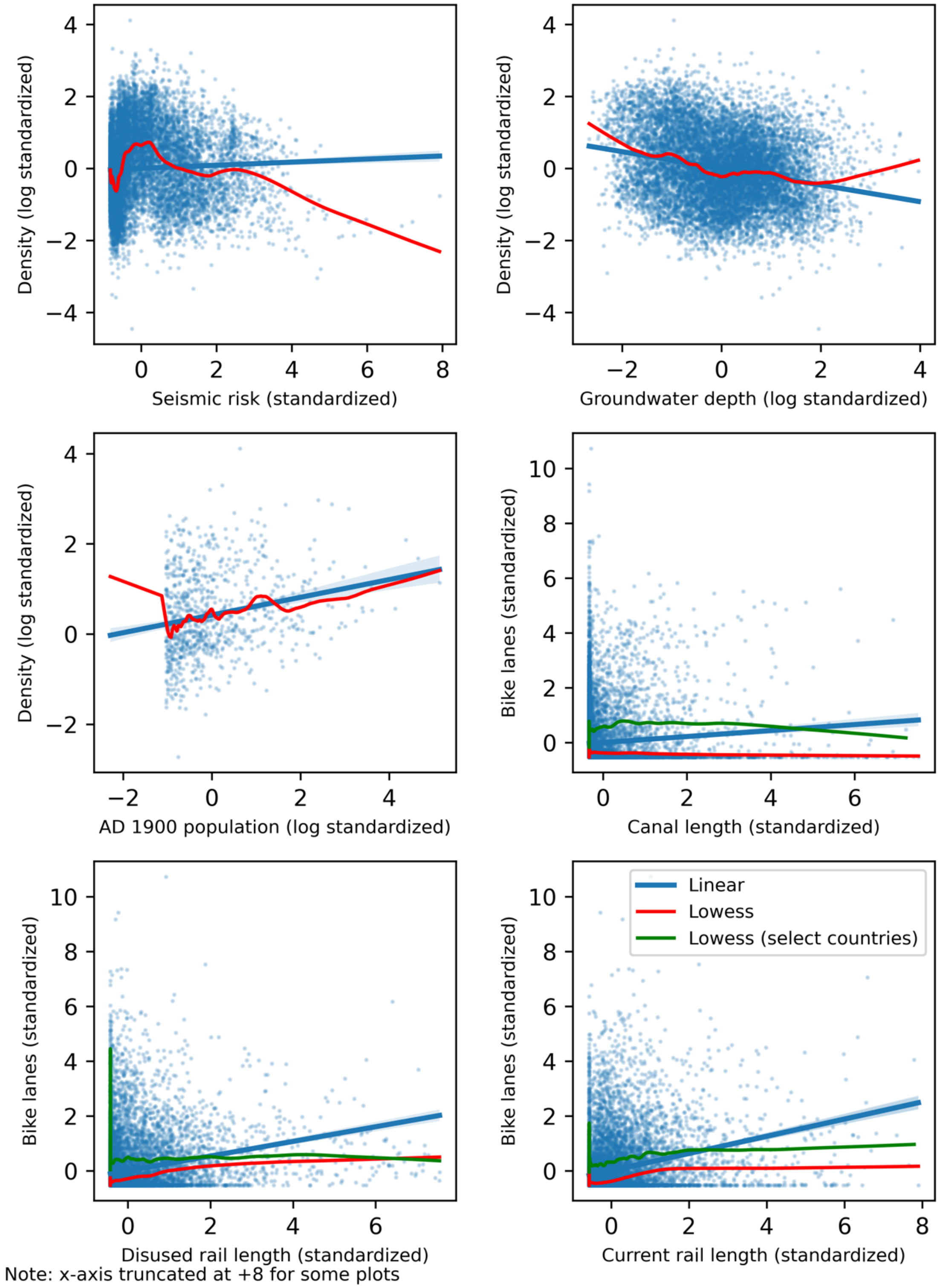

Figure SI-8. Lowess plots of the relationship between endogenous variables (density and bike lanes) and potential instruments. Bike lanes refers to the standardized km of bike lane per km of road in each city. The "select countries" lowess fit is for north American and European countries where disused rail lines and canals often provide walking/cycling routes.

	OLS	Instrumental Variables				
Instrument	N/A	Groundwater	Seismic	Soil	Historic population	All instruments
Coefficient on density: walk share	0.015*** (0.001)	0.033*** (0.008)	0.075*** (0.024)	0.038*** (0.004)	0.062*** (0.012)	0.047*** (0.008)
Coefficient on density: bike share	0.005*** (0.000)	0.022*** (0.006)	0.041*** (0.016)	0.013*** (0.003)	–0.002 (0.008)	0.005 (0.006)
N	11587	11563	11575	11580	823	821
Cragg-Donald F statistic	N/A	34.22	8.91	4.67	32.75	2.80

*** Significant at p<0.01

Table SI-3. OLS and IV estimates, using instruments for density

	OLS	Instrumental Variables		
Instrument	N/A	Canals	Disused rail	Canals + Disused rail
Coefficient on bike lanes: walk share	0.003*** (0.000)	0.025 (0.016)	–0.002 (0.092)	0.025 (0.016)
Coefficient on bike lanes: bike share	0.003***(0.000)	0.014 (0.010)	0.294 (1.110)	0.016 (0.011)
N	4566	4566	4566	4566
Cragg-Donald F statistic	N/A	12.76	0.06	6.48

*** Significant at p<0.01

Table SI-4. OLS and IV estimates, using instruments for bicycle lanes. Note that the sample is restricted to cities in Europe, the United States, and Canada.

S4. Climate and health benefits

Displacement assumptions

Our central assumption is that each new kilometer traveled by active modes displaces one kilometer that would otherwise be traveled by motorized modes (car, motorcycle, or public transportation). In effect, our 1:1 assumption implies that two countervailing effects cancel each other out. On the one hand, some walking and cycling trips are likely to be entirely new, and not displace travel that would otherwise be made by other modes. On the other hand, each walking or cycling trip may displace a longer car trip, for example, if someone walks to a nearby supermarket rather than driving to a more distant one (*30*–*32*).

The 1:1 assumption is a major uncertainty in our analysis, and the literature does not provide any definitive guidance on what the displacement ratio should be (*32*). Empirical studies typically rely on surveys which ask respondents what they would have done (e.g. driven or not made the trip) had they not walked or cycled. For example, in one study, between 63% and 96% of survey respondents (depending on the location) reported that they would have taken motorized transportation for that trip had they not walked or cycled; the average across locations was 83% (*30*).[1] Other studies are in a comparable range (e.g. *33*, *34*). But such studies have three major limitations: (i) they estimate displacement in terms of *trips* and have no information on whether displaced motor vehicle trips are the same length as the replacement walking or cycling trips; (ii) respondents are asked about a hypothetical counterfactual that they may not have considered; and (iii) studies analyze short-run effects on a per-trip basis and do not account for changes in car ownership.

More rigorous research designs using longitudinal data, randomized assignment, or similar methods for causal inference are rare. One exception is a Swedish randomized controlled trial of e-bike loans that found a displacement ratio of 1:2.1, with a cycling increase of 6.5km per day and driving reduction of 13.6km per day (*35*). However, the small sample size (N=98) and the restriction to e-bikes means that these findings may not transfer to other contexts. The second exception is a longitudinal study of travel behavior in seven European cities, which found that each additional cycling trip reduced lifecycle emissions by 0.52 kg CO_2/day, and each additional walking trip by 0.41 kg CO_2/day (*36*). The study does not report results in terms of distance, but using additional assumptions derived from the paper on trip lengths (4.8km for cycling and 1.1km for walking) and carbon intensity (0.12 kg CO_2 per passenger km by motorized modes), we derive a displacement ratio of 1:0.9 for cycling and 1:3.1 for walking.

We use these different assumptions (summarized in Table SI-5) to show the sensitivity of our estimates of greenhouse gas reductions to different displacement ratios.

---

[1] Adapted from Table 6 in Piatkowski et al. (2015). The numerator is the number of respondents who report they would have taken motorized modes (driving or public transportation). We exclude from the denominator respondents who cycled who stated that they would otherwise have walked, and vice versa, because our model treats walking and cycling jointly. We also exclude respondents who stated that they would have made the trip at a later time, since those responses give no information about a substitute mode.

Estimating CO_2 impacts

We model the impacts of increased bicycle facility provision in Stan, simulating levels of provision $B_n \in \{1, 2\ldots50\}$ km of bicycle facility per 100km of road. We take B_n as a minimum level of provision: if a city has an existing level of provision $B_0 > B_n$, its infrastructure remains unchanged at $max(B_n, B_o)$. Most cities have minimal pre-existing bicycle infrastructure (the median city has ~0.2 km of bicycle facilities for every 100km of road). Thus, our scenarios, which increase bicycle infrastructure to a given floor, would affect most cities with the exception of outliers such as Copenhagen which already have an extensive network.

We allocate the displaced travel to motorized modes depending on their pre-existing shares in each city. For example, if cars, motorcycles, and public transport each account for one-third of motorized travel, we assume that each kilometer by active modes displaces 1/3 km of car, motorcycle, and public transport travel. We convert the displaced car and motorcycle kilometers into CO_2 emission savings using country-specific fuel economy and fuel carbon content values provided in EIE.

Our main results using our central assumption for displacement are shown in Figure 5 in the main text. Table SI-5 summarizes how the results vary with different displacement assumptions.

Under our central 1:1 assumption for displacement, our estimates of emission savings per kilometer of active travel are somewhat smaller to those that use a more complex methodology to estimate displaced motorized travel. For example, Brand et al. (*36*) use a longitudinal panel in several European cities to estimate that each new walking trip saves 0.4kg CO_2, with each cycling trip saving 0.5kg. Our implied savings are 0.1 kg CO_2 per walking trip and 0.2 kg CO_2 per cycling trip. Our lower values may reflect that our estimates encompass lower-income cities where walking may displace fewer car trips and more motorcycle and public transport trips compared to the European cities analyzed by Brand et al. Moreover, our estimates are limited to in-use vehicle emissions, whereas Brand et al. quantify lifecycle emissions. It is also likely that the difference reflects the conservativeness of our method for estimating displaced trips, particularly for walking: a 1km walk trip may displace a substantial longer private vehicle trip.

A further simplification in our analysis is that we only analyze the impact of street redesign on driving through the channel of increased walking and cycling. If on-street parking is removed to install a new bicycle lane or wider sidewalk, it is plausible that fewer people would drive even if bicycle travel and walking remained constant.

We are primarily interested in emissions savings from private motor vehicle travel, and our results ignore emissions savings from displaced public transport trips. However, including them would make only a minimal difference to our results, which are expressed as percentage emission reductions. Including public transport would increase both the numerator (emission reductions) and, proportionally, the denominator (total emissions, which would now include public transportation emissions rather than just private automobiles and motorcycles). Also, even if there is substitution at the trip level, walking and cycling may be longer-term complements with public transport, for example if households reduce car ownership (*37*).

	Implied displacement ratio		CO_2 reduction scenario	
	Cycling	Walking	95th percentile	Copenhagen
Central assumption	1:1	1:1	1.9%	5.6%
Stated response surveys (*30*)	1:0.8	*	1.6%	4.9%
Randomized controlled trial (*35*)	1:2.1	*	2.5%	7.7%
Longitudinal surveys (*36*)	1:0.9	1:3.1	4.0%	11.9%

Table SI-5. Displacement ratios and estimated CO_2 reductions from private vehicles. The displacement ratio refers to passenger km of motorized travel displaced per km of walking or cycling. The 95th percentile scenario involves all cities increasing bicycle lane provision to the level of the 95th percentile city; the Copenhagen scenario, to that of Copenhagen. See Figure 5 and the associated discussion in the main text.
* No data, so the central 1:1 assumption is used

Estimating physical activity impacts

We estimate the monetary value of physical activity-related health benefits from walking and cycling based on the values in (*38*). Those values (A$0.98 per km of walking and A$0.62 per km of cycling) are given in 2016 Australian dollars (AUD). We multiply by 1.23 to convert to 2023 AUD using the Reserve Bank of Australia inflation calculator (https://www.rba.gov.au/calculator/). We then convert to 2023 US dollars using the May 9, 2024 exchange rate of 1.52. This approach is used by others such as (*39*) to approximate the health benefits of walking and cycling. As with the greenhouse gas analysis, we use Stan to simulate the mode share of walking and cycling under different values of $B_n \in \{1, 2\ldots50\}$ km of bicycle facility per 100km of road.

Note that these estimated physical activity impacts do not change with assumptions about the displacement ratio.

References

1. K. Clifton, C. D. Muhs, Capturing and Representing Multimodal Trips in Travel Surveys: Review of the Practice. *Transp. Res. Rec. J. Transp. Res. Board* **2285**, 74–83 (2012).
2. R. Buehler, J. Pucher, Overview of Walking Rates, Walking Safety, and Government Policies to Encourage More and Safer Walking in Europe and North America. *Sustainability* **15**, 5719 (2023).
3. S. Lee, J. Lee, S. H. Mastrigt, E. Kim, What cities have is how people travel: Conceptualizing a data-mining-driven modal split framework. *Cities* **131**, 103902 (2022).
4. E. Heinen, B. Van Wee, K. Maat, Commuting by Bicycle: An Overview of the Literature. *Transp. Rev.* **30**, 59–96 (2010).
5. C. Barrington-Leigh, A. Millard-Ball, A global assessment of street-network sprawl. *PLOS ONE* **14**, e0223078 (2019).
6. R. Ewing, R. Cervero, Travel and the Built Environment. *J. Am. Plann. Assoc.* **76**, 265–294 (2010).
7. A. Forsyth, K. J. Krizek, Promoting walking and bicycling: Assessing the evidence to assist planners. *Built Environ.* **36**, 429–446 (2010).

8. M. Espey, Gasoline Demand Revisited: An International Meta-analysis of Elasticities. *Energy Econ.* **20**, 273–295 (1998).

9. R. Buehler, J. R. Pucher, Eds., *Cycling for Sustainable Cities* (The MIT Press, Cambridge, Massachusetts, 2021).

10. R. Goel, A. Goodman, R. Aldred, R. Nakamura, L. Tatah, L. M. T. Garcia, B. Zapata-Diomedi, T. H. de Sa, G. Tiwari, A. de Nazelle, M. Tainio, R. Buehler, T. Götschi, J. Woodcock, Cycling behaviour in 17 countries across 6 continents: levels of cycling, who cycles, for what purpose, and how far? *Transp. Rev.* **42**, 58–81 (2022).

11. G. Duranton, D. Puga, The economics of urban density. *J. Econ. Perspect.* **34**, 3–26 (2020).

12. T. Tao, X. Wu, J. Cao, Y. Fan, K. Das, A. Ramaswami, Exploring the Nonlinear Relationship between the Built Environment and Active Travel in the Twin Cities. *J. Plan. Educ. Res.* **43**, 637–652 (2023).

13. C. Barrington-Leigh, A. Millard-Ball, The world's user-generated road map is more than 80% complete. *PLOS ONE* **12**, e0180698 (2017).

14. C. Ferster, J. Fischer, K. Manaugh, T. Nelson, M. Winters, Using OpenStreetMap to inventory bicycle infrastructure: A comparison with open data from cities. *Int. J. Sustain. Transp.* **14**, 64–73 (2020).

15. GIZ, "International Fuel Prices 2018/19" (2019); https://sutp.org/publications/international-fuel-prices-report/.

16. C. Barrington-Leigh, A. Millard-Ball, A high-resolution global time series of street-network sprawl. *Environ. Plan. B Urban Anal. City Sci.*, 23998083241306829 (2025).

17. A. Gelman, J. Hill, *Data Analysis Using Regression and Multilevel/Hierarchical Models* (Cambridge University Press, Cambridge, 2007.

18. B. Carpenter, A. Gelman, M. Hoffman, D. Lee, B. Goodrich, M. Betancourt, M. A. Brubaker, J. Guo, P. Li, A. Riddell, Stan: A probabilistic programming language. *J. Stat. Softw.* **76**, 1–32 (2017).

19. Stan Development Team, "Stan User's Guide, version 2.34" (2024); https://mc-stan.org/docs/stan-users-guide/.

20. J. Hong, Non-linear influences of the built environment on transportation emissions: Focusing on densities. *J. Transp. Land Use*, doi: 10.5198/jtlu.2015.815 (2015).

21. W. Zhang, D. Lu, Y. Chen, C. Liu, Land use densification revisited: Nonlinear mediation relationships with car ownership and use. *Transp. Res. Part Transp. Environ.* **98**, 102985 (2021).

22. J. D. Angrist, A. B. Krueger, Instrumental Variables and the Search for Identification: From Supply and Demand to Natural Experiments. *J. Econ. Perspect.* **15**, 69–85 (2001).

23. G. Duranton, M. A. Turner, Urban form and driving: Evidence from US cities. *J. Urban Econ.* **108**, 170–191 (2018).

24. R. Buehler, J. Pucher, Cycling to work in 90 large American cities: new evidence on the role of bike paths and lanes. *Transportation* **39**, 409–432 (2012).

25. J. Verkaik, E. H. Sutanudjaja, G. H. P. Oude Essink, H. X. Lin, M. F. P. Bierkens, GLOBGM v1.0: a parallel implementation of a 30 arcsec PCR-GLOBWB-MODFLOW global-scale groundwater model. *Geosci. Model Dev.* **17**, 275–300 (2024).

26. K. Johnson, M. Villani, K. Bayliss, C. Brooks, S. Chandrasekhar, T. Chartier, Y.-S. Chen, J. Garcia-Pelaez, R. Gee, R. Styron, A. Rood, M. Simionato, M. Pagani, Global Seismic Hazard Map, version v2023.1.0, Zenodo (2023); https://doi.org/10.5281/ZENODO.8409647.

27. L. Poggio, L. M. De Sousa, N. H. Batjes, G. B. M. Heuvelink, B. Kempen, E. Ribeiro, D. Rossiter, SoilGrids 2.0: producing soil information for the globe with quantified spatial uncertainty. *SOIL* **7**, 217–240 (2021).

28. M. Reba, F. Reitsma, K. C. Seto, Spatializing 6,000 years of global urbanization from 3700 BC to AD 2000. *Sci. Data* **3**, 160034 (2016).

29. C. Blaudin De Thé, B. Carantino, M. Lafourcade, The carbon 'carprint' of urbanization: New evidence from French cities. *Reg. Sci. Urban Econ.* **89**, 103693 (2021).

30. D. P. Piatkowski, K. J. Krizek, S. L. Handy, Accounting for the short term substitution effects of walking and cycling in sustainable transportation. *Travel Behav. Soc.* **2**, 32–41 (2015).

31. S. Handy, B. Van Wee, M. Kroesen, Promoting Cycling for Transport: Research Needs and Challenges. *Transp. Rev.* **34**, 4–24 (2014).

32. C. Brand, Active Travel's Contribution to Climate Change Mitigation: Research Summary and Outlook. *Act. Travel Stud.* **1** (2021).

33. G. M. Rowangould, M. Tayarani, Effect of Bicycle Facilities on Travel Mode Choice Decisions. *J. Urban Plan. Dev.* **142**, 04016019 (2016).

34. R. Mitra, R. A. Ziemba, P. M. Hess, Mode substitution effect of urban cycle tracks: Case study of a downtown street in Toronto, Canada. *Int. J. Sustain. Transp.* **11**, 248–256 (2017).

35. A. Söderberg F.K.A. Andersson, E. Adell, L. Winslott Hiselius, What is the substitution effect of e-bikes? A randomised controlled trial. *Transp. Res. Part Transp. Environ.* **90**, 102648 (2021).

36. C. Brand, T. Götschi, E. Dons, R. Gerike, E. Anaya-Boig, I. Avila-Palencia, A. De Nazelle, M. Gascon, M. Gaupp-Berghausen, F. Iacorossi, S. Kahlmeier, L. Int Panis, F. Racioppi, D. Rojas-Rueda, A. Standaert, E. Stigell, S. Sulikova, S. Wegener, M. J. Nieuwenhuijsen, The climate change mitigation impacts of active travel: Evidence from a longitudinal panel study in seven European cities. *Glob. Environ. Change* **67**, 102224 (2021).

37. P. A. Singleton, K. J. Clifton, Exploring Synergy in Bicycle and Transit Use: Empirical Evidence at Two Scales. *Transp. Res. Rec. J. Transp. Res. Board* **2417**, 92–102 (2014).

38. B. Zapata-Diomedi, L. Gunn, B. Giles-Corti, A. Shiell, J. Lennert Veerman, A method for the inclusion of physical activity-related health benefits in cost-benefit analysis of built environment initiatives. *Prev. Med.* **106**, 224–230 (2018).

39. S. Kraus, N. Koch, Provisional COVID-19 infrastructure induces large, rapid increases in cycling. *Proc. Natl. Acad. Sci.* **118**, e2024399118 (2021).
